# From Materials to Medical Images: Translating Perovskite‐Based X‐Ray Detectors Toward Clinical Imaging

**DOI:** 10.1002/advs.76156

**Published:** 2026-06-18

**Authors:** Sibin Wang, Yongrui Yang, Kun Zhang, Xiaojuan Lu, Hong Yin, Yali Qiao, Xiaojia Zheng, Xiao Zhang, Yanlin Song

**Affiliations:** ^1^ Department of Radiology the First Medical Center of Chinese PLA General Hospital Beijing P.R. China; ^2^ Chinese PLA Medical School Beijing P.R. China; ^3^ CAS Key Laboratory of Green Printing Beijing National Laboratory for Molecular Science (BNLMS) Institute of Chemistry Chinese Academy of Sciences Beijing P.R. China; ^4^ University of Chinese Academy of Sciences Beijing P.R. China; ^5^ Sichuan Research Center of New Materials Institute of Chemical Materials China Academy of Engineering Physics Chengdu P.R. China; ^6^ Medical Supplies Center of Chinese PLA General Hospital Beijing P.R. China

**Keywords:** direct‐conversion detectors, medical X‐ray imaging, metal halide perovskite, perovskite scintillators, photon‐counting CT

## Abstract

As an indispensable part of modern diagnosis and image‐guided therapy, medical X‐ray imaging requires detector materials that combine low‐dose operation, high spatial resolution, fast response, energy‐resolved capability, long‐term stability, and scalable integration. Metal halide perovskites are promising candidates for high‐performance and low‐cost X‐ray absorbers, because their strong X‐ray attenuation, tunable composition, defect tolerance, favorable charge transport, and low‐temperature processability enable both direct‐ and indirect‐conversion detector designs. However, clinical translation cannot be assessed only by sensitivity or detection limit. Practical medical detectors must also satisfy requirements in modulation transfer function, noise power spectrum, detective quantum efficiency, lag/ghosting, dynamic range, pixel uniformity, readout compatibility, radiation durability, and safety. These requirements define a key gap between laboratory demonstrations and practical clinical applications. This review examines perovskite‐based X‐ray detectors from a materials‐to‐medical‐images perspective. Conventional detector materials are first considered as reference platforms for defining clinical imaging requirements. Recent progress in perovskite‐based X‐ray detectors is then discussed with emphasis on their relevance to clinical imaging. Finally, the key barriers to clinical implementation are analyzed. By linking material properties, detector architectures, imaging metrics, and modality‐specific requirements, this review outlines a translation roadmap for perovskite‐based X‐ray detectors toward future low‐dose, high‐resolution, and energy‐resolved clinical imaging.

## Graphical abstract

This review examines perovskite‐based X‐ray detectors from a materials‐to‐medical‐images perspective. Recent progress in perovskite‐based X‐ray detectors is discussed with emphasis on their relevance to clinical imaging. The key barriers to clinical implementation are analyzed. By linking material properties, detector architectures, imaging metrics, and modality‐specific requirements, this review outlines a translation roadmap for perovskite‐based X‐ray detectors toward future low‐dose, high‐resolution, and energy‐resolved clinical imaging.

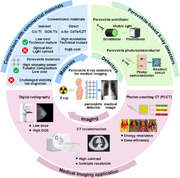

## 1 Introduction

X‐ray imaging technologies, including radiography, fluoroscopy, mammography, cone‐beam imaging, and computed tomography (CT), play a crucial role in modern clinical diagnosis [1, 2, 3]. In these systems, X‐ray detector materials strongly influence dose efficiency, spatial resolution, temporal response, and overall image quality [4, 5]. Accordingly, the continued demand for lower patient dose and higher diagnostic accuracy has made the development of detector materials a central issue in medical imaging science [6, 7].

Generally, medical X‐ray detectors are broadly divided into indirect and direct conversion systems. Indirect detectors employ a scintillator to transform X‐ray photons into visible light, which is then read out by optical sensors [8, 9]. Among these systems, CsI:Tl and Gd_2_O_2_S (GOS):Tb remain the most widely used scintillators because of their high light yield and mature device compatibility [10]. In particular, the columnar or needle‐like morphology of CsI:Tl helps restrict lateral light spread, supporting considerable spatial resolution [11, 12]. However, indirect detectors face an intrinsic trade‐off between X‐ray absorption and spatial resolution. Increasing scintillator thickness improves quantum absorption and dose efficiency, but it can also increase lateral light spreading and optical crosstalk, which reduces image sharpness [13]. Columnar CsI:Tl partially mitigates this issue by guiding scintillation light toward the photodetector, but optical blur remains an important limitation in scintillator‐based imaging systems [11]. In contrast, direct‐conversion detectors convert X‐ray photons directly into electrical charges in a photoconductor or semiconductor layer. This architecture avoids the intermediate visible‐light conversion step and is therefore attractive for high‐resolution imaging and energy‐resolved detection [14, 15]. Amorphous selenium (a‐Se) is one of the most established direct‐conversion materials for digital radiography (DR) and mammography because it can be deposited over large areas and integrated with flat‐panel readout arrays [16, 17]. Nevertheless, the relatively low atomic number of selenium limits X‐ray stopping power at higher photon energies, and high electric fields are usually required for efficient charge collection [16, 17]. CdTe and CdZnTe (CZT) offer stronger X‐ray attenuation and are important for high‐energy and spectral detection, but their large‐area use is restricted by high crystal‐growth cost, nonuniformity, charge trapping, and integration complexity [18, 19]. These limitations create a strong motivation to search for new detector materials that combine high X‐ray absorption, efficient charge transport, scalable fabrication, and compatibility with medical imaging systems.

Metal halide perovskites have attracted growing attention as X‐ray detector materials because they combine several properties that are desirable for both direct and indirect X‐ray detection. The heavy constituent elements in perovskite materials provide strong X‐ray attenuation, while their tunable composition, long carrier transport length, high resistivity, defect tolerance, and low‐temperature processability support sensitive X‐ray detection and scalable device fabrication [5, 20, 21, 22]. Since the early demonstration of direct X‐ray detection using solution‐processed lead halide perovskites [23], the field has rapidly expanded, including single crystals [20, 21, 24, 25], thick films [5, 26], wafers [27, 28], and scintillator formulations [9, 29, 30]. Perovskites are especially attractive because they can serve as direct‐conversion semiconductors, scintillation materials for indirect imaging, or absorbers for energy‐resolved radiation detection [31, 32, 33, 34].

Recent progress shows that perovskite X‐ray detectors are moving beyond single‐pixel proof‐of‐concept devices toward imaging‐oriented systems [22, 26, 35]. Thick perovskite films have been integrated with complementary metal oxide semiconductor (CMOS) or thin‐film transistor (TFT) readout arrays for radiography and CT reconstruction [26, 32, 35]. Perovskite scintillators and structured scintillator arrays have also shown promise for low‐dose, high‐resolution, and dynamic X‐ray imaging [29, 36, 37]. These advances indicate that perovskite X‐ray detection is no longer only a materials‐chemistry problem. It is increasingly becoming a system‐level challenge involving absorber thickness control, defect management, dark‐current suppression, pixel uniformity, readout integration, image‐quality metrics, and application‐specific clinical requirements.

Despite rapid progress, the further application of perovskite‐based X‐ray detectors in medical imaging is still limited by several critical challenges. Many perovskite X‐ray detector studies emphasize sensitivity and dose‐rate detection limit, which are important but insufficient for judging clinical relevance. A detector with high sensitivity may still be unsuitable for medical imaging if it exhibits strong lag, unstable dark current, poor pixel uniformity, limited dynamic range, insufficient radiation durability, or low detective quantum efficiency under realistic imaging conditions [21, 38, 39, 40]. For medical imaging, detector performance must be assessed across material, device, and system levels. Material‐level parameters include X‐ray attenuation, density, effective atomic number (*Z*_eff_), resistivity, mobility‐lifetime (*μτ*) product, light yield, and decay time. Device‐level parameters include sensitivity, detection limit, dark current, response speed, lag/ghosting, and pixel‐to‐pixel uniformity. System‐level parameters include modulation transfer function (MTF), noise power spectrum (NPS), detective quantum efficiency (DQE), dynamic range, dose efficiency, array stability, and compatibility with clinical readout electronics [41, 42, 43, 44].

**FIGURE 1:**
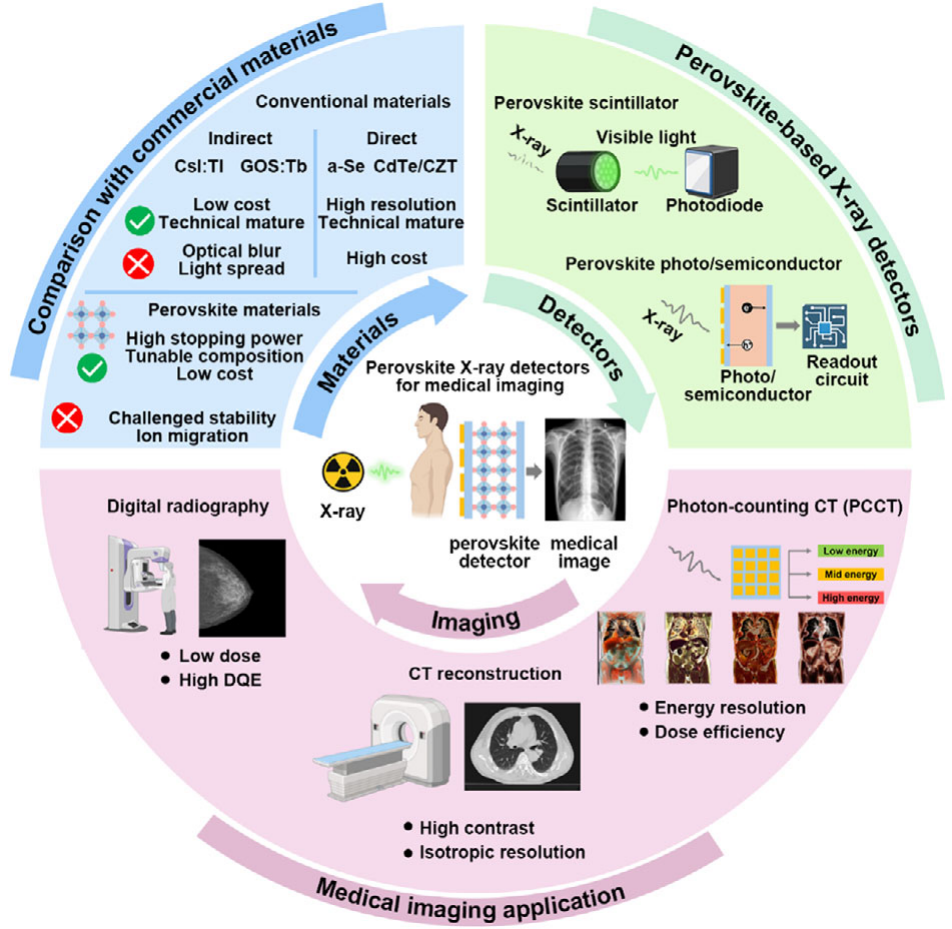
Overview of the materials‐to‐medical‐images framework of this review.

In this review, as illustrated in Figure 1, we discuss perovskite‐based X‐ray detectors through a clinical‐translation framework. First, we introduce the main detector architectures, including indirect conversion, direct conversion, energy‐integrating detection, and photon‐counting detection, together with clinically important figures of merit such as sensitivity, detection limit, MTF, NPS, DQE, lag, dynamic range, and operational stability. We then use conventional detector materials, including CsI:Tl, GOS:Tb, a‐Se, CdTe, and CZT, as clinical reference platforms to clarify where perovskites offer realistic advantages and where established materials remain superior. Subsequently, we review perovskite scintillators for indirect X‐ray imaging, including nanocrystal composites, lead‐free systems, and structured scintillator arrays. We then discuss direct‐conversion perovskite semiconductors and photoconductors in the forms of single crystals, wafers, and thick films, with emphasis on charge transport, dark‐current control, scalable thick‐absorber fabrication, and readout integration. In addition, we analyze energy‐resolved and photon‐counting perovskite detector concepts, while distinguishing laboratory single‐photon sensitivity from the demanding requirements of clinical photon‐counting CT and nuclear medicine imaging. Finally, we summarize the key challenges that must be addressed before clinical translation, including ion migration, bias‐induced polarization, dark‐current drift, environmental instability, lead safety, large‐area uniformity, CMOS/TFT integration, and standardized medical imaging evaluation.

## 2 Detector Architectures and Clinically Relevant Figures of Merit

### 2.1 Direct and Indirect Conversion Mechanisms

Medical X‐ray detectors are commonly classified by the physical pathway through which incident X‐ray photons are converted into an electronic image signal. In indirect‐conversion detectors, X‐rays are first absorbed by photo‐active materials, so‐called scintillators, and transformed into visible photons, which are then detected by a photodiode readout array [45, 46]. In direct‐conversion detectors, the X‐ray photons are absorbed in a photoconductor or semiconductor layer and converted directly into electron‐hole pairs that are collected by integrated circuits under an applied electric field [47, 48, 49]. Indirect conversion remains highly attractive for medical imaging because it is compatible with large‐area detector manufacturing and mature flat‐panel readout technologies [50, 51]. In this architecture, scintillators such as CsI:Tl and GOS‐based screens are used to provide efficient X‐ray absorption and visible‐light emission. The central design trade‐off is that increasing scintillator thickness generally improves quantum absorption and dose efficiency, but usually worsens spatial resolution due to the lateral light spread. For the most successful and widely developed columnar CsI screens, the columnar morphology helps channel scintillation light and suppresses lateral optical diffusion relative to granular materials, allowing thicker absorbing layers without a proportional loss of sharpness [52]. Direct‐conversion detectors eliminate the intermediate optical step, avoiding light‐scattering losses in scintillator‐based systems. For the design of X‐ray detectors based on direct‐conversion materials, the absorber must combine sufficiently high X‐ray stopping power with high resistivity, efficient charge generation, and carrier transport that remains adequate over clinically relevant thicknesses [22, 53, 54]. These requirements make the choice of absorber material especially stringent, since leakage current, defect density, charge trapping, and field stability directly influence both image noise and long‐term operational reliability.

Besides classification by X‐ray conversion pathway, X‐ray detectors can also be categorized by signal‐acquisition mode, most notably into energy‐integrating detectors (EIDs) and photon‐counting detectors (PCDs) [42, 55]. EIDs sum the total deposited signal over the exposure window and remain the dominant architecture in conventional digital radiography and most standard CT systems [42, 56]. By contrast, photon‐counting detectors register individual photons and sort them by energy threshold, preserving spectral information of the incident X‐ray photons. PCDs enable multi‐energy imaging, improved weighting of photon information, and better rejection of electronic noise, which are used in the advanced photon‐counting CT (PCCT) systems [57, 58]. However, these advantages are limited by challenges such as pulse pile‐up at high flux, charge sharing between neighboring pixels, and degradation of energy fidelity when count rates become too high [57, 58].

### 2.2 Material and Device Metrics Relevant to Medical Imaging

**FIGURE 2:**
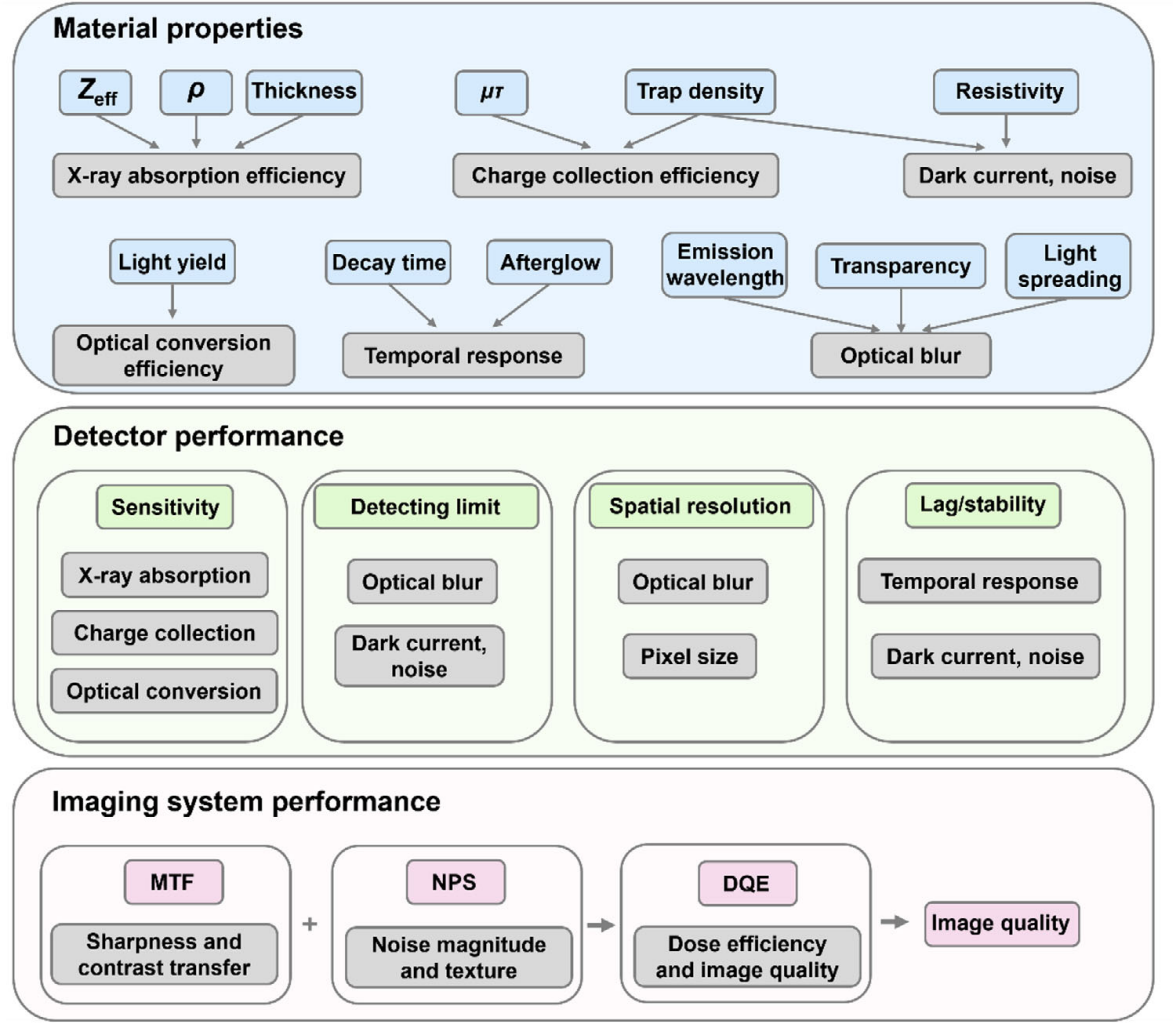
Relationship between material properties, detector‐level performance, and imaging‐system metrics in X‐ray detection. Material properties determine X‐ray absorption, charge collection, optical conversion, temporal response, optical blur, and dark‐current noise, which collectively affect sensitivity, detection limit, spatial resolution, lag/stability, MTF, NPS, DQE, and final medical image quality.

As summarized in Figure 2, for medical X‐ray imaging, the performance of a detector should be evaluated at the material level, detector level, and system level [59]. At the material level, the *Z*_eff_, density (*ρ*), and absorber thickness determine X‐ray absorption efficiency, which sets the upper limit of signal generation and dose utilization [22, 53]. However, absorber thickness is also a trade‐off parameter. In scintillator‐based indirect detectors, increasing thickness improves X‐ray absorption but can enhance lateral light spreading and optical blur [39, 60, 61]. In direct‐conversion detectors, thicker absorbers improve stopping power but require sufficiently high carrier *μτ* product and stable electric‐field distribution to avoid charge trapping, delayed response, and incomplete charge collection [62]. A good X‐ray imaging material should combine strong X‐ray stopping capability with efficient signal generation and transport. For direct detectors, this requires high attenuation, high effective atomic number and density, high resistivity, and large *μτ* product, whereas for scintillators it mainly requires strong X‐ray absorption together with high light yield and fast decay.

At the detector level, important figures of merit include sensitivity, dark current, detection limit, response time, lag/ghosting, and pixel‐to‐pixel uniformity, which reflect the practical signal quality and stability of the device [31, 63, 64]. Sensitivity describes the electrical output generated per unit X‐ray exposure or dose rate, and the detection limit defines the minimum dose rate that can be distinguished from the dark signal under a specified statistical criterion and integration time. These quantities are reported as core indices in recently reported detectors with emerging materials, such as perovskite [14, 20, 29], organic semiconductors [65, 66, 67, 68], and metal organic frameworks [69]. However, these parameters should not be overinterpreted. A detector may show very high sensitivity because of internal gain or favorable test conditions while still underperforming in terms of lag, uniformity, or DQE [63, 70]. Detection‐limit values depend strongly on how dark current and noise are modeled and measured, which makes performance less comparable across publications unless experimental conditions are carefully standardized [6].

At the system level, the most relevant figures of merit are MTF, NPS, DQE, dynamic range, and long‐term operational stability, which determine the final image quality under clinical conditions [41, 59, 71]. MTF is the standard frequency‐domain description of spatial resolution and expresses how efficiently image contrast is preserved as spatial frequency increases [43]. NPS describes the magnitude and distribution of image noise across different detail scales, indicating whether the image looks grainy or smooth [72]. DQE is a spatial‐frequency‐dependent metric derived from the detector MTF and NPS under a defined incident air kerma/fluence, and it is widely used as a surrogate measure of detector dose efficiency in radiographic imaging [73]. As summarized in Table 1, the performance of X‐ray detector materials for medical imaging should not be evaluated by a single figure of merit. Digital radiography and CBCT require large‐area detector panels with low‐dose response, high pixel uniformity, broad dynamic range, and stable MTF/NPS/DQE performance [12, 44]. Mammography places stronger emphasis on high spatial resolution and low‐noise imaging at low X‐ray energies, making direct‐conversion perovskites potentially attractive if dark‐current drift and operational stability can be controlled [74]. Fluoroscopy and interventional imaging require fast temporal response and low lag because delayed signal and baseline drift can accumulate during repeated real‐time exposures [44, 50]. Conventional CT requires high X‐ray absorption, fast response, high dynamic range, radiation hardness, and stable detector calibration under repeated high‐flux exposure [75, 76]. Photon‐counting CT further requires individual photon discrimination, high count‐rate capability, stable energy thresholds, and suppression of pulse pile‐up and charge sharing [58, 77]. Although perovskite detectors have demonstrated promising single‐photon sensitivity and energy‐resolved response, most current demonstrations remain far from the flux, pixel density, and long‐term stability required for clinical PCCT. Nuclear medicine and γ‐ray imaging also require strong radiation stopping power and energy resolution, but their detector requirements differ from diagnostic X‐ray imaging because isotope emission energy, timing response, and detector‐module integration become more important [78].

**TABLE 1:** Detector requirements for medical X‐ray and radiation imaging.

Imaging modality	Key detector requirements	Important evaluation metrics	Relevance for perovskite‐based detectors
Digital radiography	Large‐area coverage, low‐dose operation, stable signal response, sufficient spatial resolution, broad dynamic range	Sensitivity, detection limit, MTF, NPS, DQE, dynamic range, pixel uniformity	Perovskite thick films, wafers, and scintillator screens
Mammography	High spatial resolution, high DQE at low dose, low image noise, excellent detector uniformity, minimal lag	MTF, DQE, NPS, dark current, lag, pixel pitch	Direct‐conversion perovskite screens
Fluoroscopy imaging	Fast temporal response, low lag/ghosting, high frame rate, radiation durability, stable baseline under repeated exposure	Response time, lag, frame rate, dark‐current drift, DQE, operational stability	Fast perovskite scintillators and low‐bias direct detectors
Cone‐beam CT	Frame‐to‐frame stability, low lag, uniform detector response, adequate spatial resolution, low‐dose operation	Lag, MTF, NPS, DQE, pixel uniformity, dynamic range	Perovskite flat‐panel imagers have shown CT reconstruction potential
Conventional CT	High X‐ray stopping efficiency, fast response, high dynamic range, radiation hardness, detector calibration stability	Temporal response, dynamic range, DQE, radiation stability, uniformity	Still require stronger evidence of high‐flux operation
Photon‐counting CT	High count‐rate capability, energy discrimination, low electronic noise, minimized pulse pile‐up and charge sharing, stable thresholds	Count rate, energy resolution, threshold stability, charge sharing, DQE	Perovskites show early single‐photon and spectral‐response potential, but clinical PCCT requires much higher flux tolerance, pixel‐level electronic integration, and long‐term calibration stability
Nuclear medicine/γ‐ray imaging	High γ‐ray stopping power, energy resolution, timing response, radiation hardness, detector‐module integration	Energy resolution, timing resolution, count rate, detection efficiency	Potential application in γ‐ray spectroscopy and nuclear imaging concepts

## 3 Perovskite‐Based X‐Ray Detecting Materials for Medical Imaging

Medical X‐ray imaging needs detector materials that can absorb X‐rays efficiently and convert them into stable image signals with low noise and high spatial resolution. At present, various materials such as CsI:Tl, GOS:Tb, a‐Se, CdTe, and CdZnTe have been widely studied and applied in clinical X‐ray detectors, which provide mature standards for evaluating emerging materials. Metal halide perovskite materials have recently attracted strong interest because they combine high X‐ray absorption, tunable composition, good optoelectronic properties, and printable deposition processes [14, 26, 33, 79, 80]. As summarized in Table 2, currently used clinical detector materials each present distinct advantages and trade‐offs. CsI:Tl and GOS:Tb remain dominant in indirect‐conversion imaging because of their mature manufacturing routes and reliable flat‐panel integration, whereas a‐Se provides high intrinsic spatial resolution for direct‐conversion mammography systems [13, 81]. CdTe and CdZnTe are particularly attractive for spectral and photon‐counting imaging because of their strong X‐ray attenuation and favorable charge‐transport properties, although their large‐scale crystal growth and integration remain costly and challenging [76, 82]. Compared with these established materials, metal halide perovskites provide a unique combination of strong X‐ray stopping power, defect tolerance, composition tunability, and low‐temperature scalable processing. Importantly, perovskites can function as both scintillators and direct‐conversion semiconductors, making them highly versatile for future low‐dose and energy‐resolved imaging systems. However, unlike commercial detector materials, perovskites still face substantial challenges in ion migration, environmental stability, operational reliability, and large‐area uniformity, which currently limit their clinical translation [21, 83].

**TABLE 2:** Parameters of representative materials for X‐ray detection.

Material	Detector type	*Z*_eff_	Merit	Limitation	Clinical maturity	Reference
CsI:Tl	Indirect	∼54	High stopping power; columnar growth reduces optical blur	Optical crosstalk; limited energy resolution	Commercially mature for DR and CBCT	[13, 81]
GOS:Tb	Indirect	∼61	High stopping power; low‐cost powder screen; robust processing	Stronger lateral scattering than CsI:Tl; lower resolution at large thickness	Commercially mature for DR and CBCT	[13]
a‐Se	Direct	34	High intrinsic spatial resolution; large‐area TFT compatibility	Lower stopping power; high electric field; lag/trapping	Commercially mature for mammography and DR	[17, 91]
CdTe	Direct	∼50	High stopping power; good spectral resolution; energy‐resolved detection	High crystal cost; charge trapping; polarization; integration difficulty	Clinically emerging in spectral and photon‐counting CT	[75, 76]
CdZnTe	Direct	49–50	High stopping power; good spectral resolution; energy‐resolved detection	High cost; limited wafer size; nonuniformity; polarization; integration difficulty	Clinically emerging in photon‐counting CT and nuclear imaging	[76, 82]
ABX_3_ perovskites	Direct and indirect	45–67	High stopping power; tunable composition; defect tolerance; low‐temperature and printable processing	Ion migration; dark‐current drift; moisture/thermal instability; limited clinical validation	Emerging research stage	[21, 83]

Perovskite scintillators and direct‐conversion perovskite photoconductors represent two complementary routes for X‐ray imaging rather than two competing solutions. Perovskite scintillators are attractive because they can be coupled with mature photodiode, CMOS, CCD, or TFT readout systems, and their solution‐processable nanocrystals, composite films, and structured arrays have shown promising light yield, fast radioluminescence decay, and high spatial resolution when optical crosstalk is effectively suppressed [29, 84, 85, 86, 87]. Therefore, they may provide a more practical route for indirect radiography, dynamic X‐ray imaging, dental imaging, and CBCT‐related applications [37, 88]. However, their imaging performance is still limited by optical scattering, self‐absorption, afterglow, light‐extraction efficiency, and environmental stability. In contrast, direct‐conversion perovskite photoconductors avoid the intermediate visible‐light conversion process and can reduce optical blur, improve charge‐based signal collection, and potentially enable higher DQE and energy‐resolved or photon‐counting detection [21, 33, 38, 89, 90]. This makes them especially attractive for high‐resolution radiography, mammography, flat‐panel CT, and future spectral imaging. Nevertheless, direct detectors impose stricter requirements on absorber thickness, carrier *μτ* product, resistivity, dark‐current suppression, electrode interfaces, and electric‐field stability. Ion migration, bias‐induced polarization, dark‐current drift, and pixel‐to‐pixel nonuniformity remain major barriers for clinical use. Therefore, the selection between scintillator‐type and photoconductive perovskite detectors should depend on the target imaging modality. Scintillators are more compatible with existing indirect imaging platforms, whereas photoconductors offer greater long‐term potential for direct, high‐DQE, and energy‐resolved medical imaging if stability and large‐area array integration can be solved.

### 3.1 Comparison Between Perovskites and Widely Used X‐Ray Detecting Materials

#### 3.1.1 Scintillators for Indirect Detection

In commercial medical X‐ray imaging, indirect‐conversion detectors are widely used because they are technologically mature, suitable for large‐area flat‐panel fabrication, and readily compatible with established medical imaging systems. In these detectors, a scintillator layer first converts X‐ray photons into visible light, which is then collected by photodiodes, CMOS sensors, CCDs, or thin‐film transistor arrays [9, 11, 12, 29]. CsI:Tl and GOS:Tb are two representative scintillator materials for medical X‐ray imaging [11, 12, 52, 92]. CsI:Tl is attractive because it has good scintillation efficiency and can form a columnar structure that helps guide light toward the photodetector. This columnar structure reduces lateral light spread and helps maintain spatial resolution when the scintillator layer is relatively thick. GOS:Tb is commonly used as a powder scintillator screen and is valued for its practical processability and established use in flat‐panel detectors [13, 92]. However, powder‐type GOS:Tb screens suffer from stronger lateral light scattering than columnar CsI:Tl screens, which may reduce spatial resolution when the screen thickness is increased. The main advantage of scintillator‐based detectors is their technological maturity. Their main limitation is optical blur, because the visible light generated inside the scintillator can spread before it reaches the photodetector. Reflective separators, optical guiding structures, or pixelated architectures can reduce optical crosstalk, but thicker scintillators still involve a trade‐off between dose efficiency and spatial resolution. Therefore, conventional scintillator design always requires a balance between X‐ray absorption and spatial resolution [13].

Compared with commercial scintillators, perovskite scintillators contain heavy atoms such as Pb, Cs, Br, I, Bi, Ag, or In, which can provide strong X‐ray absorption [29, 84]. Their composition and crystal structure can also be adjusted to tune the emission wavelength, light yield, decay time, and stability [45, 93]. In addition, perovskite scintillators can be processed by printable methods at low temperature, which makes them attractive for low‐cost, scalable, and flexible detectors [5, 94, 95]. Lead‐free double perovskites and CsPbBr_3_‐based nanocrystal systems have shown promising performance for low‐dose real‐time X‐ray imaging, and recent studies further indicate their potential for high‐speed X‐ray imaging [45, 94, 95, 96]. However, compared with CsI:Tl and GOS:Tb, perovskite scintillators are still less mature. The main challenges lie in moisture and thermal instability, possible self‐absorption, nonuniform nanocrystal dispersion in scalable films, which must be addressed before they can compete with commercial scintillators in medical X‐ray imaging.

#### 3.1.2 Semiconductors or Photoconductors for Direct Detection

Direct‐conversion detectors use a semiconductor or photoconductor to convert X‐ray photons directly into electron‐hole pairs. The generated charges are collected by electrodes under an electric field. Direct detectors do not need the intermediate visible‐light conversion step used in scintillator detectors, which reduces optical blur and is attractive for high‐resolution medical imaging. In clinical practice, amorphous selenium (a‐Se) is one of the most established direct‐conversion materials for medical flat‐panel imaging [17, 97]. a‐Se can be deposited over large areas and integrated with thin‐film‐transistor arrays, which makes it widely used for digital radiography and mammography. Since charge carriers are collected mainly along the electric‐field direction rather than optical diffusion, a‐Se‐based flat‐panels present high intrinsic spatial resolution [98]. However, a‐Se has relatively low X‐ray stopping power at higher X‐ray energies due to the lower atomic number of selenium. a‐Se also requires a high electric field for efficient charge collection, and charge trapping can cause image lag or reduced temporal performance [98, 99]. CdTe and CdZnTe (CZT) are important high‐Z semiconductor materials for direct X‐ray detection [54]. These materials have stronger X‐ray absorption than a‐Se and can operate at room temperature. Because of the high *μτ* and resistivity, CdTe and CZT are served as crucial X‐ray absorber for 3D reconstruction imaging and energy‐resolved X‐ray detection. However, high‐quality CdTe and CZT crystals are difficult and costly to grow over large areas. Defects, charge trapping, polarization, and pixel‐to‐pixel nonuniformity can also limit their detector performance and manufacturing yield. The large‐area applications of CdTe and CZT are still limited by cost, crystal quality, and complex integration.

Compared with a‐Se, CdTe, and CZT, perovskite semiconductors/photoconductors offer a balance between X‐ray absorption and cost‐effective fabrication. Similar to CdTe and CZT, heavy elements in metal halide perovskites provide much stronger X‐ray attenuation than a‐Se. In addition, high‐quality perovskite single crystals show large *μτ*, high resistivity, and efficient charge collection, which are important for sensitive direct X‐ray detection [22, 100]. Compared with CdTe and CZT, perovskites can be processed by solution growth, coating, printing, or sintering, making them potentially more suitable for large‐area and flexible detector fabrication [64]. The single crystals, wafers, and thick films of perovskite materials provide multiple routes for different detector designs [27, 101]. However, perovskite semiconductors still face crucial challenges. The practical application of perovskite‐based direct detectors is limited by ion migration, bias‐induced polarization, dark‐current drift, and environmental instability [21, 38]. Therefore, perovskite semiconductors/photoconductors may bridge the gap between the easy large‐area processing of a‐Se and the strong X‐ray absorption of CdTe/CZT, but further improvements in stability, reproducibility, and device integration are still required before they can be used in clinical X‐ray imaging.

### 3.2 Perovskite Scintillators for Indirect X‐Ray Imaging

#### 3.2.1 Nanocrystal and Composite Scintillators

**FIGURE 3:**
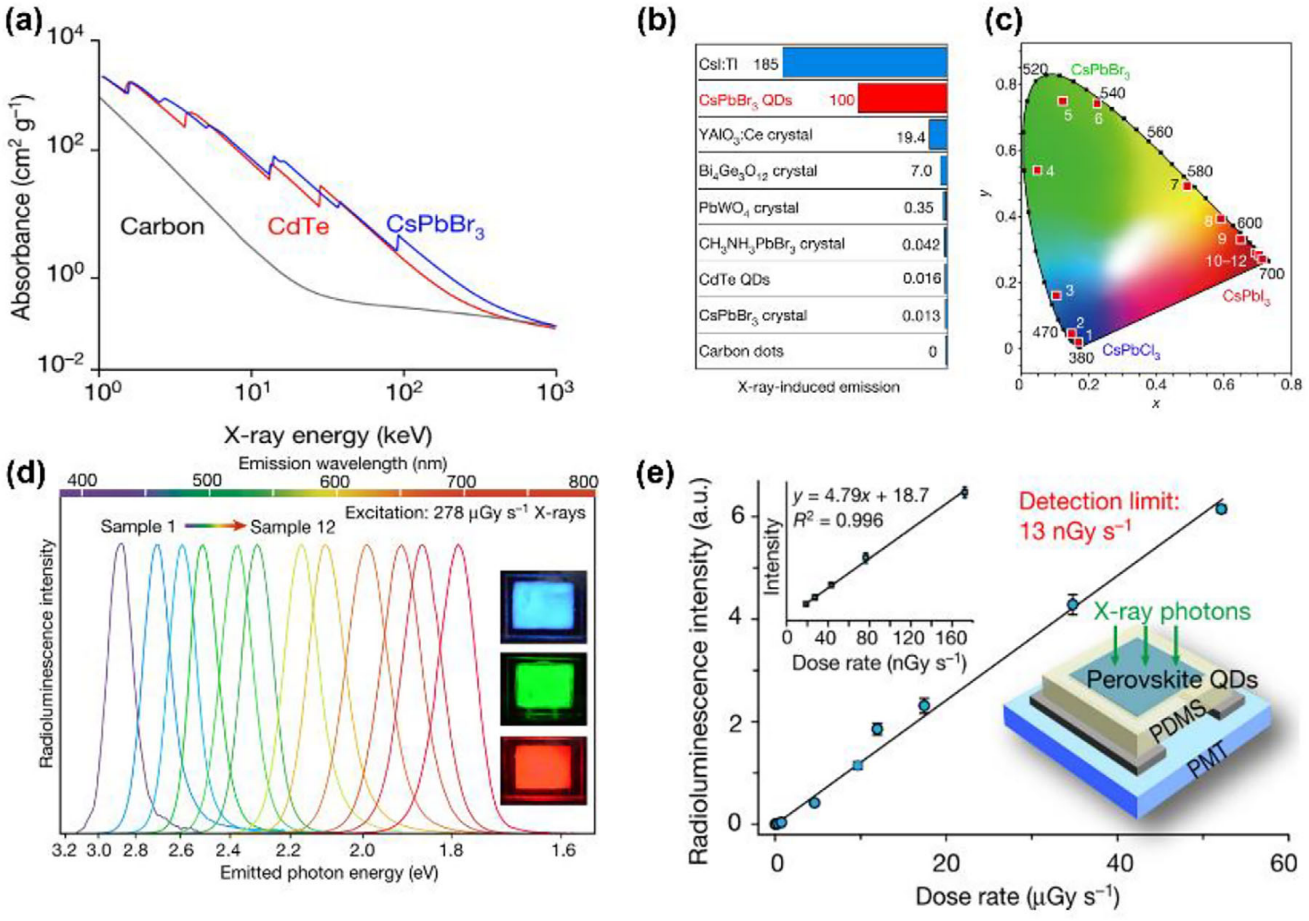
(a) Measured absorption spectra of CsPbBr_3_, CdTe, and carbon as a function of X‐ray energy. (b) Comparison of the optical sensitivity of various scintillator materials. (c) Chromaticity diagram of the X‐ray‐induced visible emissions of CsPbX_3_ nanocrystals. (d) Tunable luminescence spectra of the perovskite nanocrystals under X‐ray illumination. (e) Radioluminescence measurements for a CsPbBr_3_‐based scintillator as a function of dose rate. Reprinted with permission from ref. [29]. Copyright 2018, Springer Nature.

Perovskite nanocrystals are attractive scintillator materials because they can exhibit bright emission, fast decay, and solution‐processable fabrication [29, 93, 95]. CsPbBr_3_ nanocrystals are among the most studied perovskite nanocrystals for X‐ray scintillation. Chen et al. reported one of the pioneering studies that introduced all‐inorganic CsPbX_3_ perovskite nanocrystals as X‐ray scintillators [29]. As presented in Figure 3a,b, CsPbBr_3_ exhibits strong X‐ray absorption and bright visible emission under X‐ray excitation, which is comparable to CdTe and CsI:Tl. Traditional bulk scintillators require high‐temperature crystal growth, and their X‐ray‐induced emission color is difficult to tune. In contrast, CsPbX_3_ nanocrystals can be prepared by low‐temperature solution methods, and their radioluminescence can be tuned across the visible region by changing the halide composition (Figure 3c,d). They further fabricated flexible scintillator‐based X‐ray detectors with a very low detection limit of 13 nGy s^−1^, about 400 times lower than typical medical imaging dose rates (Figure 3e). This study opened an important direction for perovskite nanocrystal scintillators. Recently, Hu et al. developed a polar‐solvent synthesis strategy for scalable perovskite nanocrystal scintillators aimed at fast X‐ray imaging [37]. By using a low‐temperature polar‐solvent method, the authors achieved a high reaction yield of 162 mg mL^−1^ and improved exciton routing, which increased the Stokes shift and shortened the radioluminescence decay time to 7.19 ns. Benefiting from these improvements, the scintillator achieved high‐speed X‐ray imaging at 7680 frames per second with a spatial resolution of 27.6 lp mm^−1^.

**FIGURE 4:**
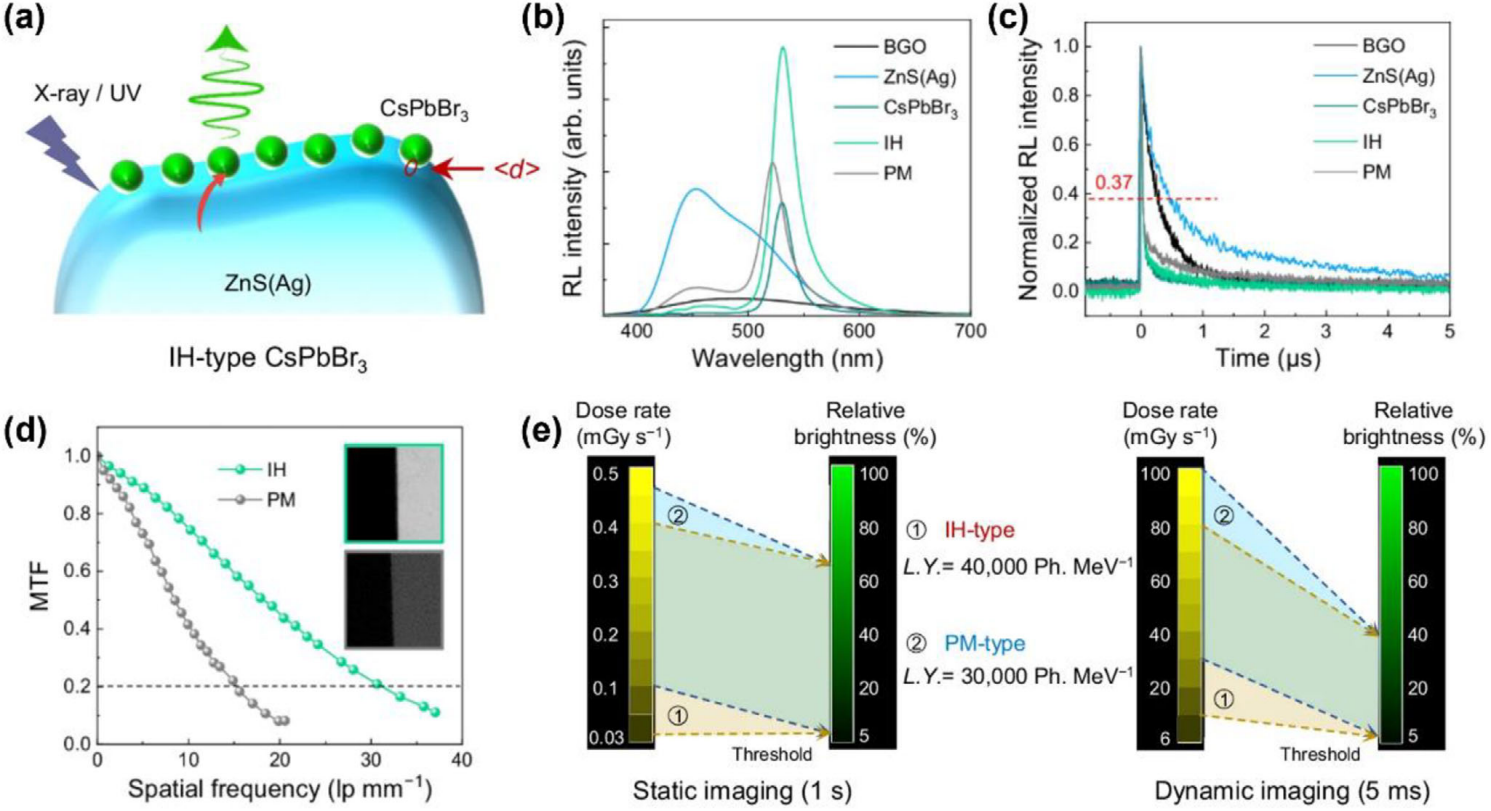
(a) Schematic of IH‐type CsPbBr_3_ structural configuration. (b) The radioluminescence spectra of scintillators under continuous X‐ray excitation. (c) Time‐resolved radioluminescence decay of scintillators under the pulse X‐ray excitation. (d) MTF curves of X‐ray detectors. (e) Relationship between the relative brightness and X‐ray dose rate of scintillators operated at static and dynamic imaging. Reprinted with permission from ref. [30]. Copyright 2024, under the Attribution‐NonCommercial‐NoDerivatives 4.0 (CC BY‐NC‐ND 4.0).

Nanocrystal scintillators can be dispersed into polymers or combined with other scintillating materials to form flexible or composite scintillator screens [36, 102, 103]. Recently, as shown in Figure 4a, Yang et al. developed a ZnS(Ag)‐CsPbBr_3_ heterostructured nanocrystal scintillator for high‐speed X‐ray imaging [30]. This work addressed the self‐absorption problem of CsPbBr_3_ nanocrystals by using ZnS(Ag) as an external energy donor and promoting efficient Förster resonance energy transfer to CsPbBr_3_. As a result, the heterostructured scintillator combined high brightness and fast response, giving a light yield of 40 000 photons MeV^−1^, a radioluminescence decay time of 36 ns, and a spatial resolution of 30 lp mm^−1^ at MTF of 0.2 (Figure 4b–d). The detectors further achieved dynamic X‐ray imaging at 200 frames per second. As presented in Figure 4e, the detectors required a much lower dose rate at both static and dynamic X‐ray imaging, suggesting that rational heterostructure design is an effective strategy for developing bright and fast perovskite nanocrystal scintillators for real‐time medical X‐ray imaging.

To address the environmental risk associated with the Pb component in perovskite materials, lead‐free perovskite scintillators are developed by replacing Pb with Bi, Sb, In, Cu, and other B‐site ions. Recently, Yang et al. developed lead‐free 3D/0D Cs_2_NaLuCl_6_/Cs_3_LuCl_6_(Sb) metal halide heterostructures for X‐ray scintillation [30]. The 0D phase provides localized exciton emission, while the 3D phase promotes charge transfer to enhance radioluminescence. Compared with the single 0D scintillator, the 3D/0D heterostructure shows 1.8‐times higher radioluminescence intensity. More importantly, the broad, spectrally flat white emission enabled color‐integrated X‐ray imaging with improved discrimination of low‐density objects. A large‐area free‐standing scintillator screen with a diameter of 16 cm was fabricated, giving a spatial resolution of 14 lp mm^−1^, good irradiation stability under an accumulated dose of 1000 Gy, and 80% radioluminescence retention at 150°C. Although lead‐free perovskite scintillators offer lower toxicity, larger Stokes shifts, and potential stability advantages, their light yield, decay speed, transparency, and imaging performance are still generally less competitive than those of Pb‐based perovskite scintillators.

#### 3.2.2 Structured Scintillator Arrays for High‐Resolution Imaging

In conventional indirect detectors, lateral light spread is one of the main reasons for reduced spatial resolution [104, 105]. To address this issue, structured scintillator arrays are applied to reduce optical crosstalk between neighboring pixels [104, 105]. Pixelated or structured scintillator arrays can confine scintillation light and improve image sharpness [87, 106]. Recently, Song et al. developed an anti‐scattering CsPbBr_3_ perovskite scintillator array by embedding CsPbBr_3_ nanocrystals into a polyurethane acrylate matrix [86]. Owing to the refractive‐index contrast between the scintillator and matrix, scintillation photons were confined within individual array pixels, which suppressed lateral light scattering and improved light collection efficiency. The device enabled low‐dose CT imaging and 3D tooth reconstruction with a spatial resolution of 20.1 lp cm^−1^ at an effective dose of 0.22 mSv, demonstrating that pixelated perovskite scintillator arrays are promising for high‐resolution medical CT imaging. Similarly, Shao et al. reported a capillary manganese halide needle‐like array scintillator. An aluminum‐clad capillary waveguide structure isolated light crosstalk and achieved very high spatial resolutions of 60.8 and 51.7 lp mm^−1^ for 0.5 and 1 mm thick scintillators, respectively [85]. Zhang et al. reported an integrated copper‐halide activated scintillator fiber array by using glass fibers embedded with Cs_3_Cu_2_X_5_ nanocrystals to construct an active fiber array of about 1600 pixels, achieving high‐resolution X‐ray imaging with 48 lp mm^−1^ resolution and a reported limit of 60.7 lp mm^−1^ [107]. Structured perovskite scintillator arrays are promising for high‐resolution X‐ray imaging because pixel isolation, optical confinement, and anti‐scattering designs can reduce lateral light crosstalk and improve image sharpness. However, their practical application is still limited by complex microfabrication, imperfect pixel uniformity, insufficient transparency and light extraction, and scalable integration with large‐area medical imaging panels, which still requires further investigation.

### 3.3 Perovskite Semiconductor/Photoconductor for Direct X‐Ray Imaging

Direct‐conversion perovskite X‐ray detectors use perovskite materials as X‐ray absorbing semiconductors [5, 21, 26]. After X‐ray absorption, electron‐hole pairs are generated inside the perovskite layer and are collected as electrical signals. The performance of these detectors depends on X‐ray absorption, resistivity, dark current, carrier mobility, carrier lifetime, trap density, interface quality, and device architecture.

#### 3.3.1 Single‐Crystal Detectors

Perovskite single crystals are attractive absorbers for direct X‐ray detection because they have fewer grain boundaries and lower defect densities, which promise longer carrier lifetimes, larger carrier diffusion length, higher *μτ*, and more efficient charge collection. These features make perovskite single crystals promising candidates for low‐dose direct X‐ray detection, high‐resolution imaging, and energy‐resolved detection [26, 38].

**FIGURE 5:**
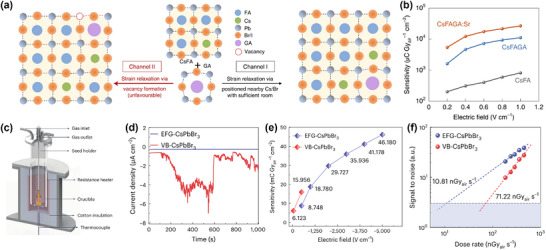
(a) Schematic of the local tensile strain in CsFAGA crystal. (b) Sensitivity of X‐ray detectors under different electric fields. Reprinted with permission from ref. [94]. Copyright 2022, under the CC BY 4.0. (c) Schematic of the atmosphere‐controlled edge‐defined film‐fed growth system. (d) Dark‐current drifts of detectors under a bias of −1000 V. (e) Sensitivities of detectors. (f) Dose‐rate‐dependent SNR of the devices. Detection limits were extracted from the fitting line at an SNR of 3. Reprinted with permission from ref. [21]. Copyright 2024, Springer Nature.

Since the pioneering work applying MAPbBr_3_ single crystal as X‐ray absorber reported by Wei et al. in 2016 [108], a main research direction for single‐crystal‐based perovskite X‐ray detectors is to grow perovskite single crystals with higher quality. During direct X‐ray detection, defects, vacancies, and lattice strain can increase dark current, reduce charge collection efficiency, and weaken operational stability [22, 109, 110]. Jiang et al. further improved perovskite single‐crystal quality through synergistic strain engineering, as presented in Figure 5a [110]. A‐site alloying is used to reduce trap density, while B‐site doping is applied to release the microstrain caused by A‐site alloying. The resulting detector showed a high sensitivity of 2.6 × 10^4^ µC Gy_air_^−1^ cm^−2^, a detection limit of 7.09 nGy_air_ s^−1^, and stable operation for more than half a year (Figure 5b). This study connects crystal strain control, defect reduction, low‐bias operation, and long‐term detector stability. Following this direction, Liu et al. reported inch‐sized triple‐cation mixed‐halide FAMACs perovskite single crystals for X‐ray imaging [111]. By introducing MA^+^, Cs^+^, and Br^−^ into the FAPbI_3_ lattice, the authors reduced lattice stress and vacancy defects, leading to stable dark current, high sensitivity, low detection limit, and clear X‐ray imaging. X‐ray detectors based on FAMACs single crystals showed the highest sensitivity of (3.5 ± 0.2) × 10^6^ µC Gy_air_^−1^ cm^−2^, under 40 keV X‐ray radiation and a low detection limit of 42 nGy s^−1^. This work showed that composition engineering is an effective way to improve both crystal quality and detector stability.

Besides improving crystal quality, another key issue is suppressing ion migration during operation [21, 112]. Ion migration can cause dark‐current drift, baseline instability, signal fluctuation, and image artifacts, especially under a strong electric field. Sakhatskyi et al. prepared thick and uniform MAPbI_3_ single‐crystal films directly on hole‐transporting electrodes and operated the devices at zero bias [22]. This device design reduced the need for a high external electric field and helped improve operational stability. The detector showed a noise‐equivalent dose of 90 pGy_air_, a detection efficiency of 88%, a spatial resolution up to 11 lp mm^−1^, and stable operation for more than one year. Ion migration can also be suppressed by controlling crystal growth conditions and halide defects. Hua et al. reported atmosphere‐controlled growth of CsPbBr_3_ single crystals in an Ar/HBr mixed atmosphere (Figure 5c) [21]. Compared with conventional vertical Bridgman‐grown CsPbBr_3_, the atmosphere‐controlled crystals showed lower trap density, higher resistivity, higher ion‐migration activation energy, reduced leakage current, and smaller baseline drift (Figure 5d). The corresponding X‐ray detector showed a sensitivity of 46 180 µC Gy_air_^−1^ cm^−2^, a detection limit of 10.81 nGy_air_ s^−1^, and stable response for 30 days (Figure 5e,f).

After crystal‐quality improvement and ion‐migration suppression, the next challenge is to design device configurations that can work at low bias and can be connected reliably with readout circuits [27, 28, 110]. Self‐powered or zero‐bias structures are especially attractive for perovskite single‐crystal detectors because they reduce dark current and weaken electric‐field‐driven ion migration [4, 22, 113]. Besides the abovementioned work reported by Sakhatskyi et al. [22], Liu et al. further developed thin perovskite monocrystals in a p‐i‐n device structure of ITO/PTAA/perovskite/C_60_/BCP/Au [114]. The resulting self‐driven detector worked without applied bias and showed sensitivity of 1.74  ×  10^5^ µC Gy^−1^ cm^−2^ and detection limit of 11.8 nGy s^−1^. Reliable interconnection between perovskite single crystals and readout circuits is another key issue for medical imaging. A single‐crystal absorber must be electrically connected to pixelated electrodes or readout circuits without damaging the crystal or introducing large contact resistance, leakage current, or pixel nonuniformity [28, 35, 115]. Wei et al. applied low‐temperature molecular bonding to integrate hybrid perovskite single crystals onto a heterogeneous substrate [116]. The bonding molecule formed both mechanical and electrical connections between the perovskite and silicon substrate, reduced device noise, and enabled perovskite pixel and linear‐array X‐ray imaging. These studies show that future perovskite single‐crystal detectors should not only focus on crystal quality, but also on device structures, stable electrode contacts, pixel‐level interconnection, and compatibility with CMOS or TFT readout circuits.

The development of perovskite single‐crystal X‐ray detectors follows a clear route, which is growing higher‐quality crystals, suppressing ion migration and dark‐current drift during operation, and then developing large‐area, thin‐crystal, and integrated device structures. Perovskite single crystals offer strong X‐ray absorption and excellent charge transport, but their practical use in medical imaging is still limited by slow crystal growth, difficult control of crystal size and thickness, surface and interface defects, and integration with large‐area pixelated readout arrays.

#### 3.3.2 Wafer‐Based Detectors

Perovskite wafers provide a balance between single crystals and films. Compared with bulk single crystals, wafers are easier to prepare with controllable thickness and larger area, and they can be fabricated by isostatic pressing, thermal pressing, sintering, or other ceramic‐like processing [53, 117, 118]. These methods are more suitable for scalable detector fabrication than slow single‐crystal growth. Compared with thin films, wafers can be made hundreds of micrometers to millimeters thick, which is useful for absorbing medical X‐rays efficiently. More importantly, wafer processing allows the use of multicomponent compositions, solid additives, grain‐boundary passivation, 2D/3D heterostructures, and wafer‐level heterojunctions, which are difficult to realize in conventional bulk single crystals [119, 120, 121]. Therefore, perovskite wafers are promising for large‐area, low‐dose, and flat‐panel direct X‐ray imaging, although their grain boundaries and interparticle interfaces can also introduce charge trapping, dark‐current drift, and ion migration.

**FIGURE 6:**
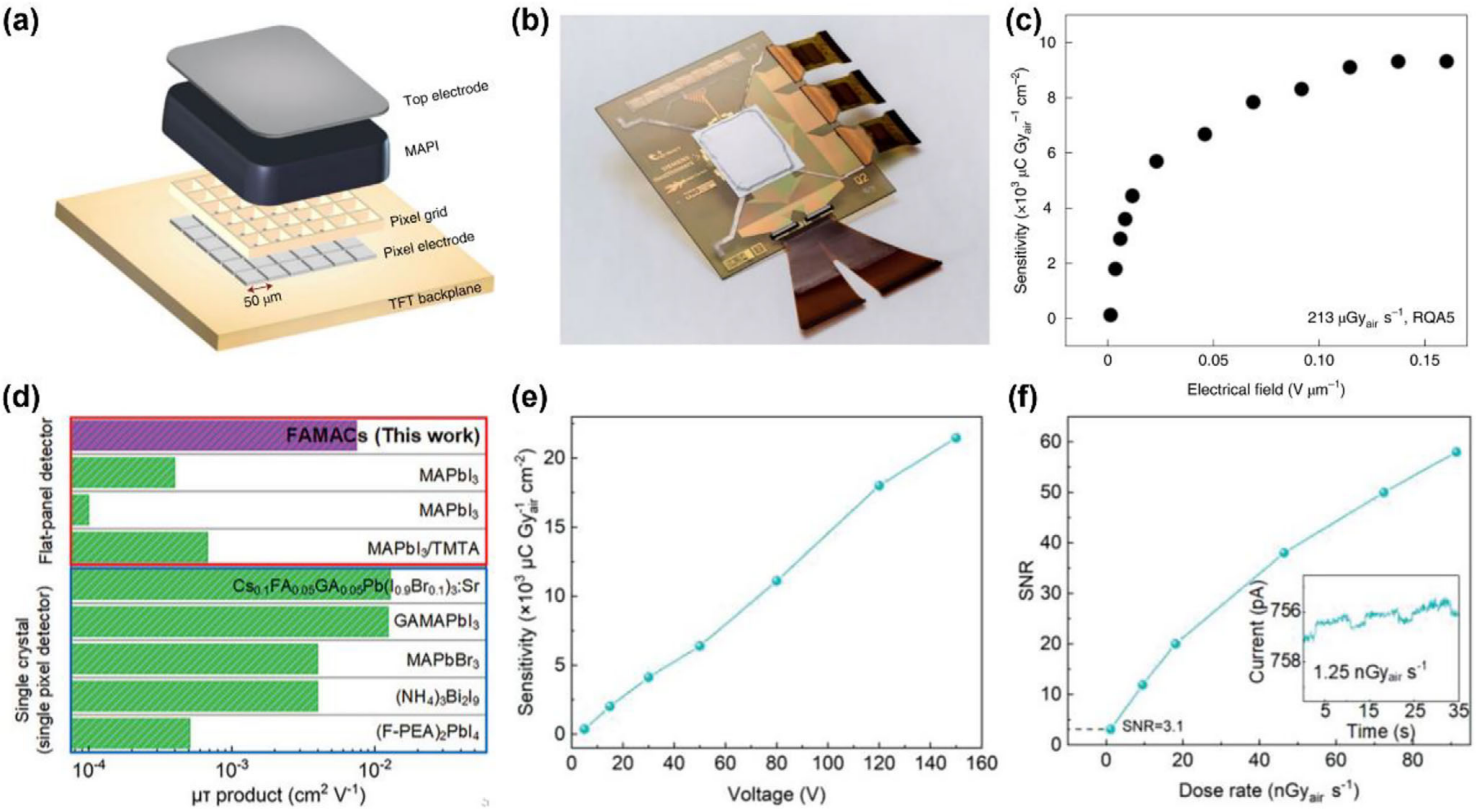
(a) Schematic of the X‐ray imager. (b) Photograph of the final detector with chip‐on‐glass gate driver integrated circuits. (c) Sensitivity of the X‐ray detector. Reprinted with permission from ref. [27]. Copyright 2021, Springer Nature. (d) Comparison of *µτ* product for perovskite single crystals and wafers. (e) Sensitivity of X‐ray detectors. (f) Dose‐rate‐dependent SNR of the devices. Reprinted with permission from ref. [126]. Copyright 2023, John Wiley & Sons.

Similar to single crystals, the first key direction is to fabricate high‐quality wafers with compact microstructure and reduced defects. Deumel et al. developed a freestanding MAPbI_3_ absorber wafer by mechanical soft‐sintered processing and then integrated onto a pixelated backplane at room temperature (Figure 6a) [27]. This two‐step process separates the absorber formation and readout integration, avoiding the thermal limitations of the electronic backplane. As presented in Figure 6c, the freestanding MAPbI_3_ wafers showed a sensitivity of 9300 µC Gy_air_^−1^ cm^−2^ and a *μτ* product of 4 × 10^−4^ cm^2^ V^−1^. After integration with a 508‐pixels‐per‐inch backplane (Figure 6b), the detector achieved 6 lp mm^−1^ spatial resolution and a low detection limit of 0.22 nGy_air_ per frame. Besides soft‐sintered processing, Liu et al. introduced PbI_2_‐DMSO solid additives into MAPbI_3_ wafers [122]. During hot pressing, DMSO vapor promoted in situ crystal growth inside the wafer, leading to higher crystallinity, lower defect density, a dense microstructure, a *μτ* product of 8.70 × 10^−4^ cm^2^ V^−1^, a sensitivity of 1.58 × 10^4^ µC Gy_air_^−1^ cm^−2^, and a detection limit of 410 nGy_air_ s^−1^. Tan et al. used nano‐sized MAPbBr_3_ powders and low‐temperature hot pressing to form transparent wafers through nanocrystal ordered coalescence [123]. The resulting wafers showed optical transparency above 60%, high uniformity, a sensitivity of 1.14×10^5^ µC Gy_air_^−1^ cm^−2^, and a detection limit of 149 nGy_air_ s^−1^, approaching the performance of MAPbBr_3_ single‐crystal detectors.

Since the polycrystalline nature of the perovskite wafers, more grain boundaries and internal interfaces in polycrystalline perovskite wafers serve as ion‐migration pathways, which intensifies ion migration and dark‐current drift in wafer devices. To suppress ion migration, Li et al. reported oriented 2D perovskite wafers prepared by a fast tableting strategy [124]. Bi^3+^ ions strengthened ionic bonding with I^−^ ions, which helped suppress ion migration, and the oriented wafer showed a detectable dose rate of 30 nGy s^−1^ under 120 kVp hard X‐ray irradiation with improvements in both charge collection and operational stability. Besides component engineering, additive and interface engineering are effective routes for improving the bias stability of wafer‐based detectors. More recently, Zhao et al. used 2‐bromonaphthalene as a solvent‐free passivator for CsPbBr_3_ wafers [125]. The molecule passivated interfacial defects, increased the ion‐migration activation energy to 0.56 eV, reduced dark‐current drift by about 100 times, and enabled a sensitivity of 11090 µC Gy_air_^−1^ cm^−2^ with a low detection limit of 9.41 nGy_air_ s^−1^.

Different from single crystals, perovskite wafers present great potential in multicomponent and heterojunction designs for scalable flat‐panel detectors. Wu et al. used a mechanochemical method to synthesize high‐entropy FA_0.9_MA_0.05_Cs_0.05_Pb(I_0.9_Br_0.1_)_3_ perovskite powders with accurate stoichiometry and kilogram‐scale yield [126]. As shown in Figure 6d‐f, the resulting flat‐panel detector showed a large *μτ* product of 7.5 × 10^−3^ cm^2^ V^−1^, a sensitivity of 2.1 × 10^4^ µC Gy_air_^−1^ cm^−2^, a detection limit of 1.25 nGy_air_ s^−1^. Liu et al. further reported a six‐inch high‐purity lead halide perovskite wafer prepared by a ceramic‐manufacturing‐inspired hot‐pressing technique [127]. The produced wafer materials present carrier mobility, lifetime, and defect concentration comparable to single crystals and enabled a 10 × 10 cm^2^ heterojunction wafer array with 256 × 256 pixels, giving a sensitivity of 36532 µC Gy_air_^−1^ cm^−2^ and a detection limit of 139 nGy_air_ s^−1^. This result is important because it directly addresses the scale‐up and wafer‐level heterostructure engineering required for practical imaging systems.

Reliable integration with readout circuits is the final key step for wafer‐based detectors. For the soft‐sintered MAPbI_3_ wafer, the wafer absorber was attached to a TFT backplane by using a photoresist grid as a mechanical anchor and recrystallized MAPbI_3_ as an adhesion promoter (Figure 6a) [27]. The device was then connected with chip‐on‐glass gate drivers and chip‐on‐flex readout circuits, showing a practical route for connecting thick perovskite absorbers to flat‐panel electronics. However, the same study also showed that the backplane must tolerate suitable electric fields and that further interlayer engineering is needed to reduce dark current and improve dynamic behavior. Wafer‐scale patterning is another useful integration strategy. Dun et al. demonstrated wafer‐scale photolithography‐pixeled Pb‐free Cs_2_AgBiBr_6_ perovskite X‐ray detectors, achieving a sensitivity of 19118 ± 763 µC Gy_air_^−1^ cm^−2^ and improved spatial resolution after pixelation [89].

Wafer‐based perovskite detectors provide a scalable route for direct X‐ray imaging by combining thick X‐ray absorption, adjustable composition, additive engineering, ion‐migration suppression, and readout‐circuit integration. Compared with single crystals, perovskite wafers are more suitable for large‐area fabrication and wafer‐level heterojunction design, but they still face challenges in grain‐boundary defects, ion migration, dark‐current drift, mechanical reliability, pixel‐to‐pixel uniformity, and stable interconnection with TFT or CMOS readout circuits.

#### 3.3.3 Film‐Based Detectors

Perovskite films are also attractive as absorbers for direct X‐ray detection due to the scalable deposition and direct formation on readout backplanes by printing, spray coating, blade coating, or vapor deposition. Compared with bulk single crystal and wafer, this route is easier to scale and more compatible with monolithic device fabrication and TFT arrays [26, 35, 128].

**FIGURE 7:**
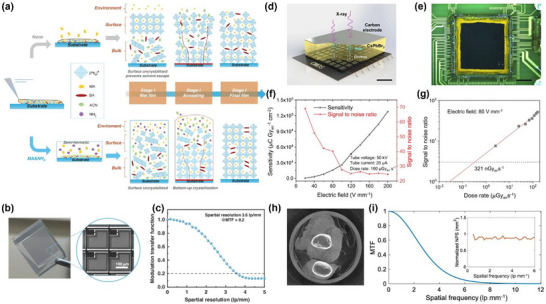
(a) Schematic of normal perovskite fabrication process and the atmosphere‐assisted process. (b) Photograph of the 64 × 64‐pixeled TFT substrate and its microstructure. (c) MTF curve of the detectors. Reprinted with permission from ref. [32]. Copyright 2024, under the CC BY 4.0. (d) Schematic of the X‐ray detector structure. The scale bar denotes 200 µm. (e) Photograph of the fabricated X‐ray detector with 6.0 mm × 6.0 mm active area. (f) Sensitivity and SNR of the detectors with respect to different electric fields. (g) The experimental lower limit‐of‐detection response curve. (h) The reconstructed CT images of a chicken drumette specimen. (i) The MTF response of the perovskite CMOS detector. Inserted plot is the NPS response of the perovskite CMOS detector. Reprinted with permission from ref. [35]. Copyright 2024, under the CC BY 4.0.

The major challenge is the deposition of high‐quality thick films with a thickness of hundreds of micrometers, which meets the requirement in direct X‐ray detectors. During various film coating methods, spray‐coating is a promising method to fabricate thick‐films. Chen et al. fabricated a thick lead‐free Cs_3_Bi_2_I_9_ perovskite film by a low‐temperature two‐step ambient spray‐coating process for direct X‐ray detection [129]. By carefully controlling the processing temperature and spray intervals, they obtained a dense thick film with well‐stacked domains, which improved charge transport and reduced dark current. The resulting detector showed a sensitivity of 127.23 µC Gy_air_^−1^ cm^−2^, a detection limit of 7.4 nGy_air_ s^−1^, and stable photoresponse under repeated X‐ray pulses. Spray coating can provide a practical route to large‐area, low‐cost, and thickness‐controllable perovskite absorbers, which is a clear processing advantage of films compared with slow‐grown single crystals and separately prepared wafers. Besides increasing film thickness, thick‐film performance can also be improved by controlling film dimensionality and interfacial structure. Xu et al. developed sequential growth of 2D/3D double‐layer perovskite films to lower the dark current and improve direct X‐ray detection [130]. The device reached a detection limit of 480 nGy_air_ s^−1^. As shown in Figure 7a, Dong et al. grew quasi‐2D perovskite thick films in a mixed methylamine/ammonia atmosphere and obtained high‐quality films with thicknesses of hundreds of micrometers [32]. This growth method improved film quality, increased the ion‐migration activation energy to 0.632 eV, which is much higher than that of MAPbI_3_ (0.301 eV), and raised the *μτ* product to 7.81 × 10^−5^ cm^2^ V^−1^. They then introduced a TiO_2_ heterojunction, which reduced the dark current by hundreds of times under reverse bias and improved the detection limit. The final detector showed an ultrahigh sensitivity of 29 721.4 µC Gy_air_^−1^ cm^−2^, a detection limit of 20.9 nGy_air_ s^−1^, and a flat‐panel imager with a spatial resolution of 3.6 lp mm^−1^ (Figure 7b,c).

For the integration with readout circuits, thick‐film perovskite absorbers present considerable compatibility with TFT backplanes. Liu et al. reported a screen‐printed CsPbBr_3_ thick film larger than 300 µm directly screen‐printed on a 72 × 72 CMOS array with 83.2 µm pixels (Figure 7d,e) [35]. Hot pressing was then applied to rearrange the grains and form a dense and compact film. The detector showed a *μτ* product of 5.2 × 10^−4^ cm^2^ V^−1^, a sensitivity of 15891 µC Gy_air_^−1^ cm^−2^ (Figure 7f), a detection limit of 321 nGy_air_ s^−1^ (Figure 7g). For medical imaging, it enabled 3D CT imaging at 300 frames per second (Figure 7h) and achieved 5.0 lp mm^−1^ radiographic resolution at a dose of 260 nGy. The nearly flat NPS curve suggests minimal signal correlation (cross‐talking) between neighboring pixels (Figure 7i). Furthermore, He et al. reported a nearly 1 mm‑thick spray‑coated perovskite absorber deposited onto a large‑area detector array to form a direct‑conversion X‑ray imager with high DQE (≈ 80 %) and a low NED of 153 pGy_air_. The nearly micron‑level surface uniformity and wafer‑scale processing enabled uniform pixel response across the array, and the detector array was used to successfully reconstruct a 3D CT image of a human tooth under a low effective dose around 5.5 µSv, about two orders of magnitude smaller than typical dental cone‑beam CT doses.

Overall, perovskite films appear to be a promising scalable route for direct medical X‐ray imaging because of their low‐cost, scalable thick‐layer fabrication and compatibility with TFT backplanes. However, thick films still face the challenges of high trap density in hundreds‐micrometer films, high dark current, ion migration, and polarization.

### 3.4 Stability and Reliability Under Clinically Relevant Operating Conditions

Although perovskite‐based X‐ray detectors have demonstrated high sensitivity, low detection limits, and promising imaging capability. Their stability under clinically relevant operating conditions remains one of the most important barriers to medical translation. For practical medical imaging, stability should not only refer to material preservation under ambient storage, but also to stable signal generation and image quality during continuous electrical bias, repeated pulsed X‐ray exposure, elevated operating temperature, accumulated radiation dose, and long‐term integration with pixelated readout electronics [22, 28, 131]. These stresses are particularly important for direct‐conversion detectors, where thick perovskite absorbers are operated under electric fields, and for dynamic imaging modalities such as fluoroscopy, CBCT, and CT, where many consecutive frames must be acquired with minimal lag and baseline drift [132, 133].

The main stability challenges can be summarized into four closely related categories: ion migration and polarization [21, 131], dark‐current drift [28, 38], environmental degradation [103, 119], and radiation‐induced or exposure‐induced instability [110]. Under continuous bias, mobile ions and vacancies can redistribute inside the perovskite absorber, causing internal electric‐field distortion, baseline drift, hysteresis, sensitivity variation, and image artifacts. This issue becomes more severe in thick films and wafers because grain boundaries, interfaces, and defects can provide ion‐migration pathways. Dark‐current drift is also critical because it increases the detector noise floor and reduces low‐dose detectability. In clinical imaging, where stable calibration and reproducible pixel response are required, dark‐current drift may directly degrade NPS, DQE, and long‐term image uniformity. Table 3 summarizes the main operating stresses, degradation mechanisms, clinical consequences, recommended evaluation metrics, and possible mitigation strategies for perovskite‐based X‐ray detectors. Clinical stability is a multidimensional requirement rather than a single aging test. For direct‐conversion perovskite detectors, continuous bias is especially challenging because electric‐field‐driven ion migration can couple with trap states, grain boundaries, and electrode interfaces, leading to dark‐current drift and signal hysteresis. For dynamic medical imaging, repeated pulsed exposure is another critical stress condition. Fluoroscopy, CBCT, and CT require many sequential frames, so delayed signal release, scintillator afterglow, persistent photoconductivity, or ion redistribution can cause lag and ghosting artifacts. These artifacts may be less obvious in single‐frame radiography but can become serious in multi‐frame reconstruction.

**TABLE 3:** Stability challenges and mitigation strategy for perovskite‐based X‐ray detectors under clinically relevant operating conditions.

Operating stress	Degradation pathway	Clinical consequence	Mitigation strategy
Continuous electrical bias	Ion migration, bias‐induced polarization, electrode reactions, field redistribution, trap‐assisted leakage	Dark‐current drift, baseline instability, sensitivity fluctuation, image artifacts	Low‐bias or zero‐bias operation, ion‐blocking layers, blocking contacts, p‐i‐n/n‐i‐p structures, 2D/3D interfaces, defect passivation
Repeated pulsed X‐ray exposure	Charge trapping, delayed charge release, persistent photoconductivity, scintillator afterglow, ion redistribution	Lag/ghosting, frame‐to‐frame instability, reduced temporal resolution	Trap passivation, fast scintillators, optimized absorber thickness, improved charge extraction, low‐defect interfaces
Elevated temperature	Phase instability, accelerated ion migration, increased leakage current, organic‐cation volatility, contact degradation	Increased noise, shortened lifetime, calibration drift	All‐inorganic or low‐dimensional compositions, thermal encapsulation, stable electrodes, interface engineering
Moisture and oxygen exposure	Hydration, surface oxidation	Loss of sensitivity, nonuniform response, reduced light yield	Encapsulation, hydrophobic ligands, polymer matrices, lead‐sequestration layers, robust lead‐free compositions
High cumulative radiation dose	Radiation‐induced defects, trap generation, chemical degradation, contact/interface damage	Sensitivity loss, dark‐current increase, energy‐resolution degradation, pixel nonuniformity	Radiation‐hard compositions, defect‐tolerant structures, stable transport layers, and post‐irradiation recovery studies
Large‐area readout integration	Radiation‐induced defects, trap generation, contact/interface damage	Defective pixels, nonuniform images, cross‐talk, and calibration difficulty	Scalable coating/printing control, planarization, patterned absorbers, interface passivation, CMOS/TFT‐compatible processing
Photon‐counting or spectral operation	Threshold drift, charge sharing, pulse pile‐up, dark‐count fluctuation, energy‐response instability	Poor energy discrimination, inaccurate material decomposition, unstable spectral imaging	High‐resistivity absorbers, fast charge collection, pixelated electrodes, optimized readout electronics, low‐noise contacts

## 4 Photon‐Counting and Energy‐Resolved Perovskite Radiation Detectors

### 4.1 Photon‐Counting Detectors and Application in Clinical CT

Compared with widely used energy‐integrating detectors, which accumulate charge over time in proportion to the total X‐ray dose, photon‐counting X‐ray detectors (PCXDs) operate by directly measuring individual X‐ray photons, offering a significant advancement over traditional energy‐integrating detectors [42, 55]. Energy‐integrating detectors integrate the total deposited signal and therefore lose photon‐energy information, particularly in low‐dose imaging, where noise becomes a significant factor. In contrast, photon‐counting detectors measure the energy and count the number of individual incident X‐ray photons, which enhances image quality by improving contrast resolution, reducing noise, and enabling energy‐resolved imaging. By classifying each photon based on its energy, PCXDs can provide better material differentiation and achieve higher spatial resolution compared to traditional energy‐integrating detectors.

In clinical practice, photon‐counting CT (PCCT) represents a transformative advancement. PCCT allows for better material differentiation, improved low‐dose imaging, and energy‐specific data acquisition [7, 42, 57]. These features are particularly important in pediatric imaging, cardiac CT, and oncology. The ability to distinguish between different materials, such as bone, soft tissue, and contrast agents, using PCCT opens up new avenues for quantitative imaging (e.g., determining tissue composition) and enables more precise treatment planning for radiation therapy. Additionally, PCCT reduces beam hardening artifacts and improves image quality at low radiation doses, contributing to patient safety and enhanced clinical outcomes.

Despite these advantages, the requirements for clinical PCCT are much more demanding than those for laboratory single‐pixel or small‐array photon‐counting demonstrations. A clinical PCCT detector must individually resolve X‐ray photons under very high flux, sort them into stable energy bins, suppress electronic noise, and maintain reliable performance over large pixelated detector arrays during repeated high‐speed CT acquisition. Medical CT can operate at extremely high X‐ray flux rates, reported up to approximately 10^9^ counts s^−1^ mm^−2^, where pulse pile‐up, count loss, and spectral distortion become major limitations if detector response and readout electronics are not sufficiently fast [55, 134]. In addition, charge sharing, fluorescence escape, Compton scattering, threshold instability, pixel‐to‐pixel nonuniformity, and radiation‐induced drift can degrade both spatial resolution and energy accuracy. Photon‐counting CT detectors must therefore count incoming photons and measure their energies reliably, usually by applying one or more energy thresholds to convert pulse‐height information into energy‐bin data. These requirements are substantially stricter than simply demonstrating single‐photon sensitivity under low‐flux conditions.

### 4.2 Current Progress in Perovskite Photon‐Counting and Energy‐Resolved Detectors

**FIGURE 8:**
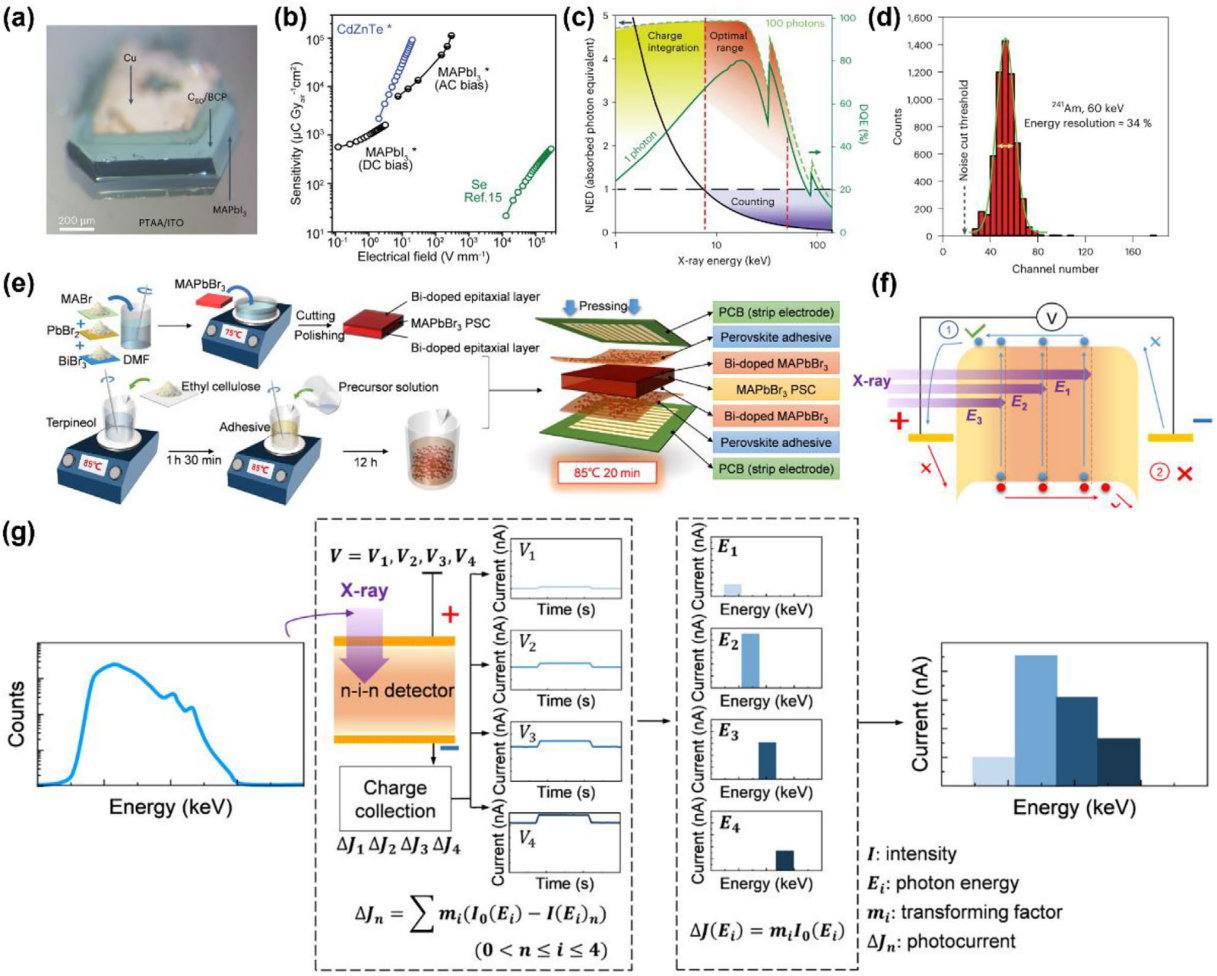
(a) Photograph of the perovskite detectors using thick and uniform MAPbI_3_ single‑crystal grown on hole‑transporting electrodes. (b) Sensitivity of the X‐ray detectors. (c) noise‐equivalent dose (NED, black line) and DQE (green lines) versus photon energy. (d) Energy‐resolved spectrum of 60 keV photons from a radioactive ^241^Am source. Reprinted with permission from ref. [22]. Copyright 2023, under the CC BY 4.0. (e) Procedure for fabricating the perovskite single crystal and the n–i–n X‐ray detector. (f) Schematic diagrams of carrier dynamics in the n–i–n X‐ray detector. (g) Proposed working mechanism for multi‐energy detection in the n–i–n X‐ray detector. Reprinted with permission from ref. [90]. Copyright 2026, under a Creative Commons Attribution NonCommercial License 4.0 (CC BY‐NC).

Significant progress has been made in the development of perovskite‐based photon‐counting detectors [135, 136, 137]. Perovskite materials have shown strong X‐ray absorption, favorable charge‐carrier transport, and potential for energy‐resolved detection, making them ideal candidates for photon‐counting X‐ray detectors. Recent studies have demonstrated the ability of perovskite materials to achieve single‐photon sensitivity and energy‐resolved detection. To resolve individual X‑ray photons, a detector material must have high X‑ray absorption (high Z and density), high resistivity to suppress electronic noise, and a large carrier *μτ* product to efficiently collect all charge generated by a single photon without loss. These properties together help lower the noise floor so that the signal from a single X‑ray photon can be distinguished from baseline fluctuations, which is essential for photon‑counting and spectral imaging. Sakhatskyi et al. demonstrated single‑photon‑counting capability and long‑term stable performance in perovskite detectors operated in photovoltaic (zero‑bias) mode by using thick and uniform MAPbI_3_ single‑crystal films grown directly on hole‑transporting electrodes (Figure 8a) [22]. This detector achieved a high X‐ray detection sensitivity of 15891 µC Gy_air_^−1^ cm^−2^, a low dose detection limit of 321 nGy_air_ s^−1^_,_ and a NED of about 90 pGy_air_ with 18 keV X‑rays (Figure 8b,c). As presented in Figure 8d the detector arrays demonstrated single‐photon counting from ^241^Am source (60 keV) with an energy resolution of ∼34%. The zero‑bias photovoltaic mode suppresses bias‑induced ion migration and enables stable energy‑resolved photon counting over extended operation. Zhou et al. reported self‑powered polycrystalline perovskite photodetectors with suppressed shallow traps and surface passivation, which significantly reduced dark count rate and enabled competitive photon‑counting performance compared with commercial silicon photomultipliers (SiPMs) [4]. Although this study is focused on optical photon counting rather than hard X‑ray counting, it illustrates how trap management and grain boundary control can dramatically improve perovskite photon‑counting sensitivity and noise performance. In addition to true photon‐counting operation, several perovskite detector concepts have demonstrated spectral or multi‐energy X‐ray imaging without resolving individual photons through conventional photon‐counting electronics. Li et al. reported a multi‐energy X‑ray detection using perovskite detectors by regulating unipolar carrier collection through the applied working voltage [90]. An n–i–n unipolar perovskite detector architecture was designed in which electrons, rather than both electrons and holes, dominate carrier transport (Figure 8e,f). As displayed in Figure 8g, by adjusting the external bias, the authors controlled the drift length of X‑ray‑generated electrons as a function of photon energy, effectively tuning the depth at which different X‑ray energies are collected. This enables multi‐energy X‑ray discrimination without requiring single‑photon counting electronics or complex multilayer structures, and the approach works under normal high‑flux imaging conditions, rather than low‑flux requirements typical of photon‑counting systems. Using a customized multi‐energy digital subtraction algorithm, the detector produced fast four‑energy‑bin X‑ray images capable of distinguishing materials that appear similar in conventional energy‑integrating images. This work showcases a voltage‑controlled spectral imaging modality that bridges the gap between energy‑integrating radiography and full photon‑counting CT by extracting energy information through carrier‑collection engineering rather than strict single‑photon counting.

Besides X‐ray detection, perovskite‐based photon‐counting detectors have also been applied in γ‑ray detection, highly relevant to medical nuclear imaging (e.g., positron emission tomography) where resolving individual γ‑ray events with high energy and spatial resolution is crucial. Shen et al. demonstrated high‑resolution perovskite CsPbBr_3_ detectors with pixelated configurations [78]. The resulting devices delivered record energy resolutions of ∼2.5% at 141 keV and ∼1.0% at 662 keV, performance on par with or exceeding conventional semiconductor detectors. Under ^99m^Tc γ‑ray sources, the detectors achieved very low count‑rate thresholds (∼0.13%–0.21% cps/Bq) and spatial resolution of ∼3.2 mm in phantom imaging, demonstrating that perovskite detectors can resolve individual γ photons with high fidelity for nuclear medicine imaging.

In summary, perovskite photon‑counting detectors have moved from material‑level demonstrations to true single‑photon‑sensitive X‑ray detectors with high detecting sensitivity and clear energy discrimination. The most typical work to date shows that thick, high‑quality perovskite absorbers in photovoltaic (zero‑bias) mode can achieve stable, low‑noise photon counting with clinically relevant spatial resolution. However, these results do not yet establish operation under the high‐flux, high‐count‐rate, multi‐threshold, small‐pixel, large‐area, and long‐term calibration conditions required for clinical PCCT. Future perovskite PCCT studies should therefore move beyond single‐pixel sensitivity and radioactive‐source spectra toward clinically relevant tests, including count‐rate linearity, pulse pile‐up tolerance, energy‐threshold stability, charge‐sharing correction, spectral DQE, defective‐pixel fraction, array uniformity, radiation durability, and repeated CT acquisition under realistic X‐ray tube spectra. This distinction is essential to avoid overestimating the current maturity of perovskite photon‐counting detectors while still recognizing their promise for future low‐cost and energy‐resolved X‐ray imaging.

## 5 Summary and Outlook

**FIGURE 9:**
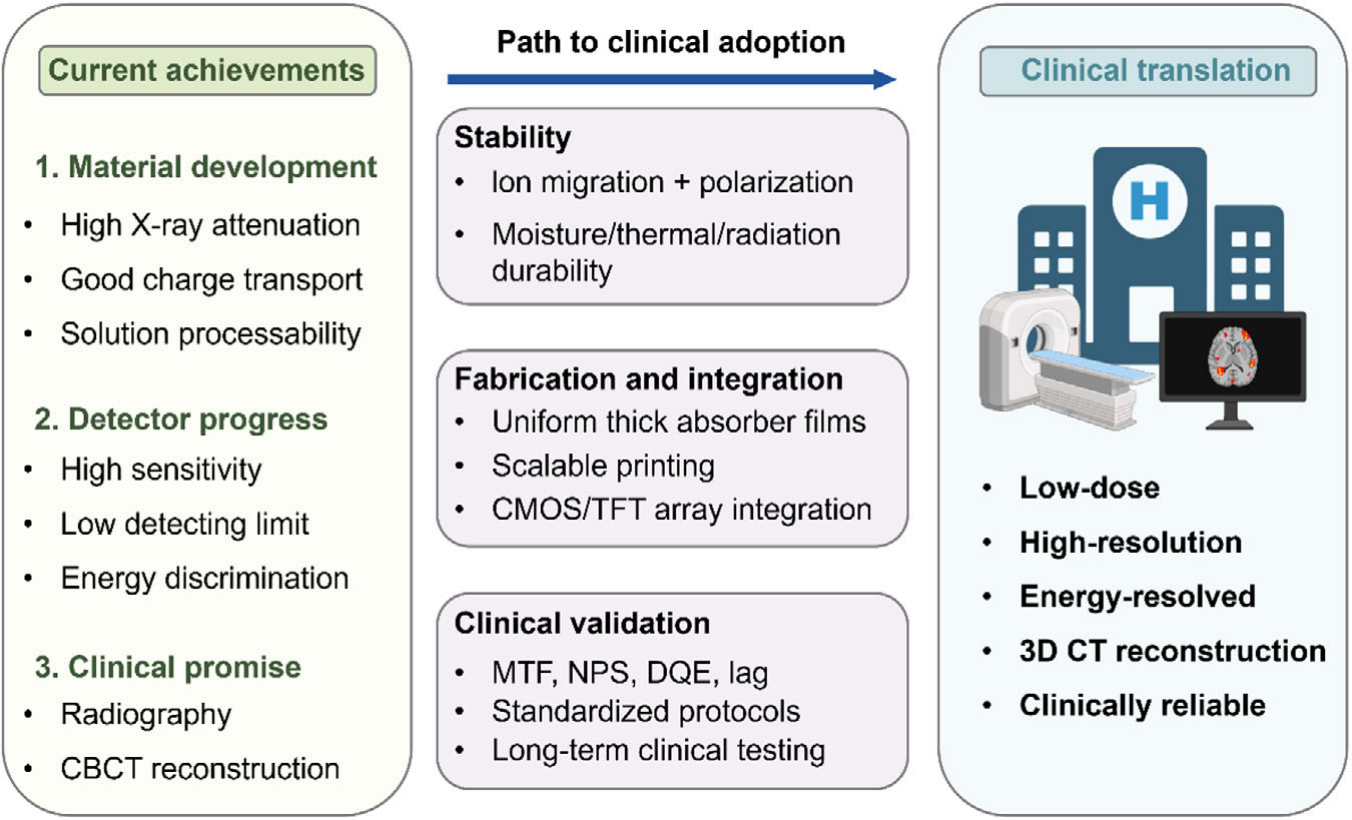
Summary and outlook for translating perovskite‐based X‐ray detectors toward clinical imaging. Certain images in the figure are created with BioRender.com.

As summarized in Figure 9, the translation of perovskite‐based X‐ray detectors toward clinical imaging can be viewed as a pathway from material development to detector engineering and finally to clinical validation. At the material level, metal halide perovskites provide several important advantages, including high X‐ray attenuation, favorable charge transport, and solution processability. These properties have enabled detector‐level progress such as high sensitivity, low detection limit, and energy discrimination in both direct‐conversion and indirect‐conversion device architectures. Together, these advances support the clinical promise of perovskite detectors for low‐dose radiography, high‐resolution imaging, CBCT reconstruction, and potentially energy‐resolved X‐ray imaging.

However, the pathway from current achievements to clinical translation still requires progress in three closely connected directions:

### 5.1 Improvement of Material Stability

Ion migration and polarization are major obstacles for perovskite detectors, particularly in thick absorber films. Future research should focus on developing new perovskite compositions, such as lead‐free and low‐dimensional perovskite structures or perovskite‐polymer composites that offer better ion migration resistance. Additionally, surface passivation techniques and heterojunctions should be further explored to reduce defects and enhance material stability. Besides ion migration, environmental stability remains a concern for perovskite‐based detectors, particularly regarding moisture, thermal, and irradiation degradation. Research into encapsulation materials and hermetic sealing technologies is crucial to improving the operational lifetime and ensuring the safety of perovskite‐based X‐ray detectors in clinical settings.

### 5.2 Scalable Fabrication and Device Integration

One of the main advantages of perovskite detectors is their scalability, especially for large‐area X‐ray imaging. However, fabricating thick, high‐quality perovskite films (hundreds of micrometers thick) while maintaining uniformity and low defects remains a challenge. Future research should focus on improving solution‐processing techniques, such as spray coating, blade coating, and screen printing, to achieve uniform, high‐quality films that can be scaled up for direct X‐ray detection in medical imaging.

Zero‐bias or self‐powered device structures are highly attractive for perovskite‐based detectors because they can reduce the impact of ion migration and improve stability. Future research should continue to explore self‐powered perovskite detectors that operate without high electric fields, improving their long‐term stability and noise performance in medical imaging applications.

For future medical imaging applications, integration of perovskite single crystals and wafers with large‐area imaging arrays will be as important as improving intrinsic material properties. The most promising strategies include direct growth of thick single‐crystal films on charge‐selective electrodes, low‐temperature bonding to silicon or TFT substrates, freestanding wafer transfer, wafer‐scale pixelation, and heterojunction wafer arrays. Future work should focus on pixel‐to‐pixel uniformity, defective‐pixel control, low‐leakage contacts, mechanical adhesion, CMOS/TFT compatibility, and standardized MTF, NPS, DQE, lag, and lifetime evaluation under clinically relevant exposure conditions.

### 5.3 Performance Metrics and Clinical Testing

While perovskite X‐ray detectors have demonstrated high sensitivity, detection limits, and spatial resolution, system‐level performance metrics like modulation transfer function (MTF), noise power spectrum (NPS), detective quantum efficiency (DQE), and long‐term operational stability must be rigorously tested under clinically relevant conditions. Research should aim to standardize evaluation protocols (such as IEC 62220‐1‐1:2015, IEC 62220‐1‐2:2007, IEC 62220‐1‐3:2008, and AAPM Report 150/TG‐150) for perovskite detectors in medical imaging, ensuring that their real‐world performance meets or exceeds the standards set by conventional materials. A practical common testing protocol for perovskite X‐ray detectors should therefore report at least the following information: X‐ray tube voltage, filtration, half‐value layer or radiation quality, source‐to‐detector distance, field size, air kerma, dose rate, integration time or frame rate, detector thickness, pixel pitch, active area, applied bias or electric field, readout electronics, and encapsulation condition. For image‐quality evaluation, studies should provide signal‐transfer response, presampled MTF, two‐dimensional or one‐dimensional NPS, DQE as a function of spatial frequency and air kerma, lag/ghosting, dark‐current drift, dose linearity, dynamic range, pixel‐response uniformity, defective‐pixel fraction, and stability after repeated pulsed exposure or accumulated radiation dose. These parameters should be reported under clinically relevant exposure conditions rather than only under optimized laboratory conditions.

Once these challenges are addressed, perovskite‐based X‐ray detectors could evolve from laboratory prototypes into clinically deployable imaging platforms that combine strong X‐ray absorption, scalable fabrication, and system‐level image‐quality performance. Stable thick absorbers integrated with CMOS/TFT readout arrays and validated by standardized MTF, NPS, DQE, lag, and lifetime protocols would enable low‐dose radiography, high‐resolution mammography, and CBCT, and energy‐resolved or photon‐counting CT with improved dose efficiency and diagnostic information. In this future, perovskite detectors are expected not only to compete with existing detector technologies, but also to expand the possibilities for safer, more accessible, and more quantitative medical X‐ray imaging.

## Author Contributions

**Sibin Wang**: conceptualization, investigation, writing – original draft, writing – review and editing. **Yongrui Yang**: conceptualization, investigation, writing – original draft, writing – review and editing, visualization, validation, methodology, formal analysis. **Kun Zhang**: writing – review and editing, formal analysis, visualization, validation. **Xiaojuan Lu**: writing – review and editing, visualization, validation. **Hong Yin**: validation, visualization, writing – review and editing. **Yali Qiao**: visualization, writing – review and editing, validation, investigation, conceptualization. **Xiaojia Zheng**: conceptualization, investigation, visualization, writing – review and editing, validation, methodology. **Xiao Zhang**: conceptualization, investigation, writing – original draft, validation, visualization, writing – review and editing, methodology. **Yanlin Song**: conceptualization, investigation, funding acquisition, writing – original draft, methodology, validation, visualization, writing – review and editing.

## Conflicts of Interest

The authors declare no conflicts of interest.

## Data Availability

The data that support the findings of this study are available from the corresponding author upon reasonable request.
